# Excision of a bulky rectal cancer with uterine invasion via a perineal approach in the right lateral decubitus position: a case report

**DOI:** 10.1093/jscr/rjaf1112

**Published:** 2026-01-20

**Authors:** Shuichiro Uchiyama, Tsuyoshi Takaya, Ichiro Niina

**Affiliations:** Kushima Municipal Hospital, Kushima, Miyazaki, Japan; Kushima Municipal Hospital, Kushima, Miyazaki, Japan; Junwakai Memorial Hospital, Miyazaki, Japan

**Keywords:** abdominoperineal resection, bulky rectal cancer, invasion of adjacent organs, the right lateral decubitus position

## Abstract

Although the lithotomy or Lloyd–Davies position is standard for laparoscopic or robotic abdominoperineal resection for lower rectal cancer, visualization and instrument maneuverability can be limited in cases involving bulky tumours with invasion of adjacent organs. We report a case of a bulky rectal cancer with uterine invasion that was successfully resected via the perineal approach, with excellent visualization achieved using the right lateral decubitus position.

## Introduction

Abdominoperineal resection (APR) has gradually been replaced by anterior resection and low anterior resection for tumours of the upper and middle rectum. However, APR remains necessary for selected cases of low rectal cancer [1]. We herein report a case of bulky rectal cancer with uterine invasion that was successfully resected via the perineum, with the vagina dissected under direct vision in the right lateral decubitus position.

## Case report

**Figure 1:**
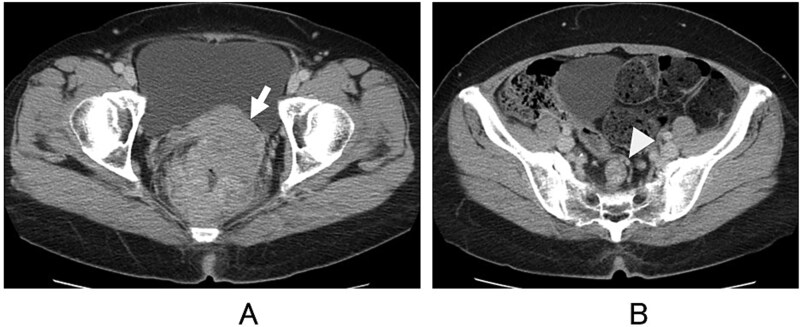
Computed tomography. Direct invasion of the uterus (A, arrow) and regional lymph node metastasis (B, arrowhead) are shown.

A 53-year-old woman presented with abdominal distension and hematochezia at a nearby hospital, where colonoscopy revealed a circumferential ulcerated tumour in the lower rectum. She was subsequently admitted to our hospital for further evaluation and treatment. Abdominal computed tomography showed localized wall thickening of the upper and lower rectum, measuring 75 × 72 × 95 mm, with uterine invasion (Fig. 1A) and two swollen regional lymph nodes (Fig. 1B). No distant metastases, including liver or lung metastasis, were detected. The proximal colon was distended due to stenosis, and a loop colostomy was created in the right transverse colon. The colonoscope could not pass beyond the tumour, and histologic examination of the endoscopic biopsy specimen confirmed tubular adenocarcinoma. Magnetic resonance imaging further clarified the uterine invasion and showed no evidence of lateral lymph node metastasis. Transvaginal examination by a gynecologist revealed no tumor invasion into the vaginal cavity.

**Figure 2:**
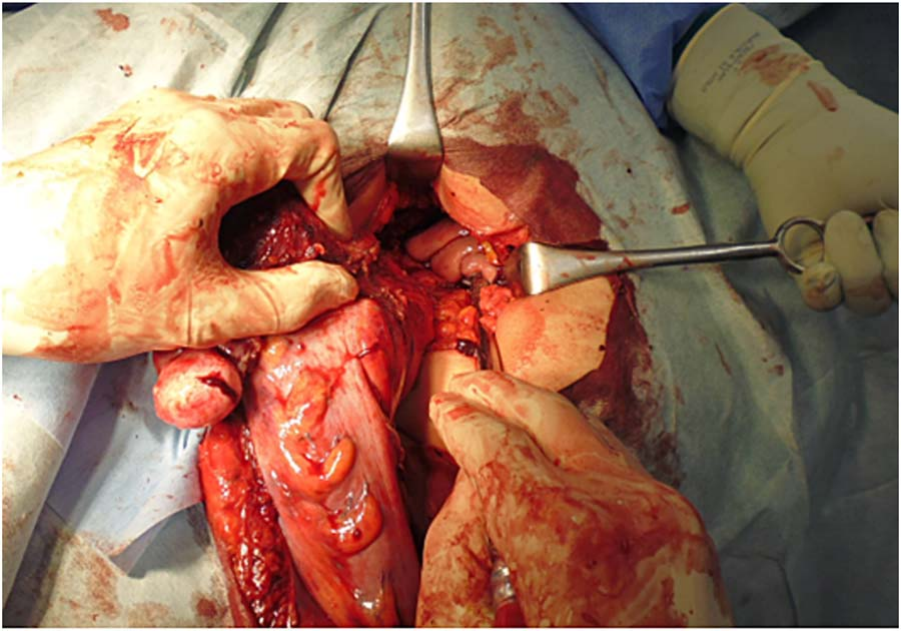
Specimen extraction. With the patient in the right lateral decubitus position, the proximal end of the colon was extracted, and the specimen remained attached to the posterior vaginal wall.

An elective surgery was planned for treatment of the rectal cancer with uterine invasion. Under general anesthesia, bilateral ureteral stents were placed by a urologist with the patient in the lithotomy (LT) position. Although the tumour compressed the ureters, there was no evidence of ureteral invasion. Laparoscopic examination confirmed that the lesion was confined to the rectum and uterus, with no findings of liver metastasis or peritoneal dissemination. The loop colostomy was closed after confirming tumour resectability. A hysterectomy was performed: after dissection of the ligaments and vessels, an incision was made in the anterior vaginal wall using a vaginal tube, while the posterior vaginal wall was preserved. Rectal dissection was then carried out: the root of the inferior mesenteric artery was dissected, and a total mesorectal excision (TME) was performed as far as possible to the pelvic floor. The oral side of the colon proximal to the tumour was transected intracorporeally, and an end colostomy was created through the extraperitoneal route. After completion of the laparoscopic procedure and wound closure, the patient was repositioned to the right lateral decubitus position to facilitate perineal resection and access to the retrorectal space. The proximal colon was extracted following dissection of the levator ani muscle, and the specimen, connected to the posterior vaginal wall, was dissected with sufficient surgical margins under direct vision (Fig. 2). The postoperative course was uneventful, and the patient was discharged 32 days after surgery.

**Figure 3:**
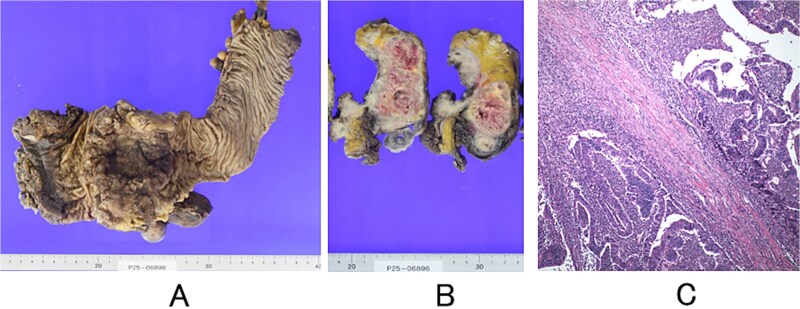
Resected specimen and histopathology. (A) Surface of the rectum after surgical removal. (B) Cross-section showing invasion of the uterus. (C) Microscopic findings showing moderately to well-differentiated tubular adenocarcinoma with direct invasion into the uterus (haematoxylin and eosin stain; original magnification, ×100).

The resected specimen showed an ulcerated lesion in the upper and lower rectum, measuring 100 × 80 mm (Fig. 3A), with direct invasion into the uterus observed on cross-section (Fig. 3B). Microscopic examination revealed atypical cells with large, hyperchromatic nuclei proliferating in irregular tubular, fused, or cribriform patterns, accompanied by fibrous stroma invading the uterus directly (Fig. 3C). Two lymph node metastases were identified, and adjuvant chemotherapy was administered.

## Discussion

**Figure 4:**
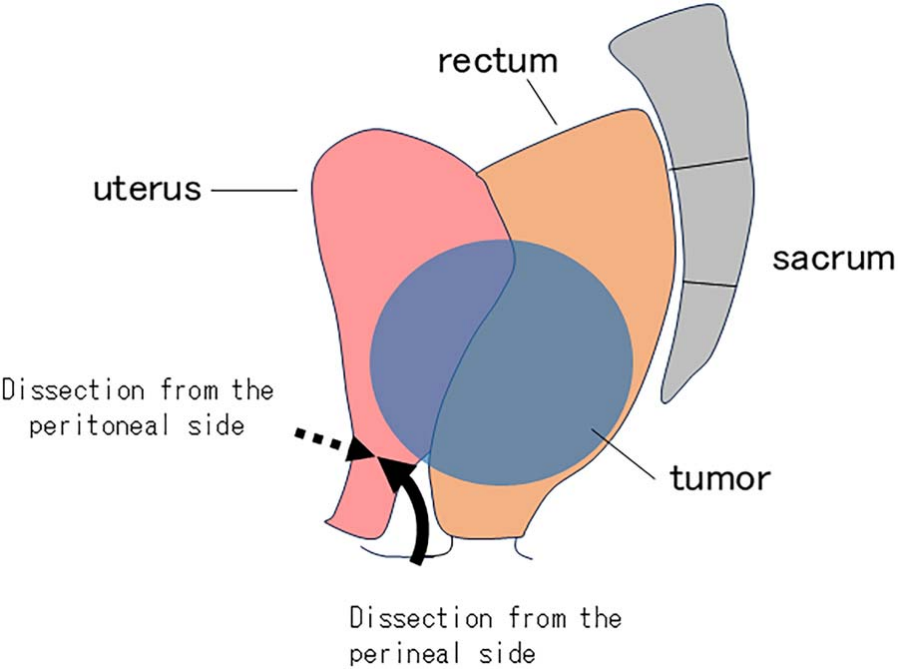
Schematic illustration of the uterine resection. Dissection from the peritoneal side (dotted arrow) and from the perineal side (solid arrow) is shown.

Several reports have evaluated the perioperative and oncological outcomes of APR performed in the prone jackknife (PJ) position compared with the classic LT or Lloyd-Davies positions. A systematic review and meta-analysis of nine studies involving 888 patients reported that the PJ position was associated with lower rates of intraoperative perforation and circumferential resection margin (CRM) involvement, as well as shorter operative times during APR [2]. Another analysis, including seven nonrandomized retrospective cohorts comprising 1663 patients, demonstrated that APR in the PJ position resulted in decreased operative time and estimated blood loss (*P* < 0.01); however, no significant differences were observed in perineal wound infections, rectal perforation, CRM positivity, or 5-year local recurrence [3]. Furthermore, a report analyzing two randomized controlled trials and 26 observational studies, including a total of 4529 patients, showed that the PJ position was associated with reduced surgical specimen perforation and lower positive CRM rates. Intraoperative bleeding was also decreased, though no significant differences were noted in operative time, urinary retention, urinary injury, wound infections, perineal dehiscence, Clavien-Dindo grade ≥ 3 complications, reoperation, local or distal recurrence, or overall survival [4]. Only one study has specifically addressed the advantage of the lateral decubitus position during the perineal phase following an abdominal phase in the LT position. In this position, the dissecting plane between the rectum and vagina or prostate is perpendicular to the surgeon’s line of sight, which decreases blood loss and operative time [5]. This approach was particularly important in our case for dissecting the posterior wall of the vagina, as the bulky tumor obscured the view during the laparoscopic procedure. A sagittal schematic illustration of the uterine resection is shown in Fig. 4.

In our practice, the anus is often excised and rectal dissection is completed in the right lateral decubitus position via a perineal approach. We adopted this method in the present case to secure a sufficient surgical margin under direct vision.

Total neoadjuvant therapy (TNT) has emerged as a standard treatment option for locally advanced rectal cancer in selected patients. Several studies suggest that TNT should be strongly considered in patients at high risk of local failure, in whom a favorable local response is desired (e.g. clinical T4 tumours, threatened or involved circumferential resection margins, lateral pelvic node involvement), or in patients at high risk of distant failure, such as those with N2 disease or extramural venous invasion [6]. Organ preservation can be achieved in approximately half of rectal cancer patients treated with TNT, without a detrimental effect on survival compared with historical controls treated with chemoradiotherapy, TME, and postoperative chemotherapy [7]. Therefore, TNT should be considered in selected cases of locally advanced rectal cancer.
